# Risk factors associated with mortality in individuals with type 2 diabetes following an episode of severe hypoglycaemia. Results from a randomised controlled trial

**DOI:** 10.1177/14791641211067415

**Published:** 2022-01-28

**Authors:** Sam M Pearson, Noppadol Kietsiriroje, Beverley Whittam, Rebecca J Birch, Matthew D Campbell, Ramzi A Ajjan

**Affiliations:** 1 Leeds Institute of Cardiovascular and Metabolic Medicine, University of Leeds, Leeds, UK; 2 Division of Diabetes and Endocrinology, 26686 Prince of Songkla University, Hat Yai, Thailand; 3 4472 Leeds Teaching Hospitals NHS Trust, Leeds, UK; 4 Leeds Institute of Medical Research at St James’s, 4468 University of Leeds, Leeds, UK; 5 Leeds Institute of Data Analytics, University of Leeds, Leeds, UK; 6 Faculty of Health Sciences and Wellbeing, 7735 University of Sunderland, Sunderland, UK

**Keywords:** Hypoglycaemia, mortality, nurse-led intervention, diabetes severity score

## Abstract

**Background:**

Severe hypoglycaemia may pose significant risk to individuals with type 2 diabetes (T2D), and evidence surrounding strategies to mitigate this risk is lacking.

**Methods:**

Data was re-analysed from a previous randomised controlled trial studying the impact of nurse-led intervention on mortality following severe hypoglycaemia in the community. A Cox-regression model was used to identify baseline characteristics associated with mortality and to adjust for differences between groups. Kaplan-Meier curves were created to demonstrate differences in outcome between groups across different variables.

**Results:**

A total of 124 participants (mean age = 75, 56.5% male) were analysed. In univariate analysis, Diabetes Severity Score (DSS), age and insulin use were baseline factors found to correlate to mortality, while HbA1C and established cardiovascular disease showed no significant correlations. Hazard ratio favoured the intervention (0.68, 95% CI: 0.38–1.19) and in multivariate analysis, only DSS demonstrated a relationship with mortality. Comparison of Kaplan-Meier curves across study groups suggested the intervention is beneficial irrespective of HbA1c, diabetes severity score or age.

**Conclusion:**

While DSS predicts mortality following severe community hypoglycaemia in individuals with T2D, a structured nurse-led intervention appears to reduce the risk of death across a range of baseline parameters.

## Introduction

Type 2 diabetes (T2D) is characterised by chronic hyperglycaemia, resulting in increased risk of vascular complications and reduced life expectancy.^1^ Treatment intensification which aims to reduce hyperglycaemia decreases risk of microvascular complications^2^ and long-term macrovascular risk.^3^ However, intensification of glycaemic treatment increases hypoglycaemic exposure, which is believed to be associated with an increased risk of cardiovascular events.^4–6^ As such, guidelines suggest individualising HbA1c targets while also focusing on hypoglycaemic avoidance and acknowledging that more research in this area is required.^7^

Recently, we conducted a randomised controlled trial (RCT) investigating the impact of a structured nurse-led intervention on mortality in people with T2D following severe hypoglycaemia. The intervention, which focused on patient education, initiation of self-monitoring of blood glucose, and pragmatic medication changes resulted in a reduction in all-cause mortality. Mean baseline HbA1c tended to be higher in those who survived till study completion compared with patients who died at any point, suggesting that outcome can be influenced by pre-treatment patient characteristics. However, the use of individual metrics in isolation, such as HbA1c, fails to capture the complexity of T2D and have limited application for risk stratification and predicting clinical outcome. Conversely, tools that combine routine clinical metrics to better capture global comorbidity and overall diabetes severity have been shown to yield greater predictive value than individual proxy indicators, including HbA1c.^8^

Therefore, the aim of this exploratory post-hoc analysis was to understand risk factors for mortality following severe hypoglycaemia in T2D and investigate possible heterogeneity in response to the intervention, which would help to identify subgroups of patients who show enhanced, or reduced, benefits from nurse-led support.

## Methods

We performed an exploratory post-hoc analysis using data from a previously published RCT^9^ (clinical trial registration NCT04422145). The RCT received ethical approval from the UK National Health Service Health Research Authority (reference 100244), and all participants gave written informed consent. Detailed information about study procedures have been published previously. In brief, patients with diabetes who suffered an episode of severe community hypoglycaemia requiring the assistance of emergency services in the area surrounding Leeds, UK, were randomised to either standard care or a structured nurse-led intervention. The intervention centred on the initiation of self-monitoring of blood glucose, education surrounding the avoidance of hypoglycaemia and adjustment of glycaemic medication, as appropriate. The main intervention took place in the 3 months following recruitment with further limited involvement of the research nurse for a further 9 months. Data surrounding mortality were collected using electronic records, and death, including cause, was confirmed using death certificates, a statutory requirement in the UK. In the present analysis, we included patients with T2D randomised to standard care and intervention arms, given that the intervention failed to show an effect on those with type 1 diabetes (T1D).

Participants were stratified into subgroups using an established Diabetes Severity Score which has previously been shown to be associated with mortality and healthcare costs.^10^ The severity score is a composite of hard clinical endpoints and biochemical variables and represents an assessment of existing, as well as risk of developing, diabetes-related complications. Scores range from 1 to 4 with 4 categorised as the greatest degree of diabetes severity. Scores were then compressed to provide dichotomous variables (groups 1 + 2 and 3 + 4). The matrix used to calculate diabetes severity score is available as Supplementary material, which is taken from the original journal article by Gibson et al.^10^

## Statistical analysis

Data analysis was performed using SPSS version 27 (IBM corporation, USA) and assessed for normality. Continuous variables are reported as mean ± SD and categorical variables reported as frequency (%). Differences between dichotomised variables were assessed using independent t-tests or chi-square test. We employed univariate and multivariate analyses using a Cox-regression model to identify factors associated with an increased risk of mortality. Only factors with *p* < 0.2 from the univariate analysis were put into the multivariate analysis for further adjustment. Statistical significance was determined as *p* < 0.05 for all analyses. Graphpad (Prism 9) USA was used to construct Kaplan-Meier curves comparing survival between groups and a logrank test used to ascertain differences in trends among curves.

## Results

### Baseline characteristics

A total of 124 participants were included in this analysis. Of these, 60 were in the intensive group and 64, the standard of care group. Of those in the intensive group, 33 (55%) were male, mean age was 74.2 (± 10.7) years and mean HbA1c was 58.5 (± 13.4) mmol/mol. Of those in the standard of care group, 37 (57.8%) were male, mean age was 74.8 (± 10.2) years and mean HbA1c was 60.0 (± 16.2) mmol/mol. Mean follow-up for the intensive study arm was 40.9 (± 15.5) months and in the standard of care arm 39.0 (± 15.9) months, which was not significantly different (t-test, *p* value = 0.52.) Additional information on baseline characteristics is available in Table 1.

**Table 1.** A summary of baseline characteristics. Continuous variables displayed as mean ± SD and categorical as number (percentage.) Independent t-test used for comparisons between means of continuous variables and chi-squared or Fisher’s exact test for comparison between categorical variables. Insufficient data was available for severity score calculation for 3 participants, 1 in the intervention group and 2 in standard of care. Established cardiovascular disease was defined as the documentation in the participants’ medical records of a history of angina, previous myocardial infarction, stroke disease (haemorrhagic or ischaemic), lower limb amputation or peripheral vascular disease requiring intervention.

	*Intensive (n = 60)*	*Standard (n = 64)*	*Difference between groups (p value)*
Male (%)	33 (55)	37 (57.8)	0.75
Age (years)	74.2 ± 10.7	74.84 ± 10.2	0.82
Presenting capillary glucose (mmol/L)	2.3 ± 0.7	2.3 ± 0.8	0.94
HbA1c (mmol/mol)	58.5 ± 13.4	60.0 ± 16.2	0.59
Body mass index (kg/m^2^)	31.0 ± 7.7	29.5 ± 5.8	0.20
Duration of diabetes (years)	21.4 ± 11.3	20.2 ± 11.1	0.55
Established cardiovascular disease (%)	21 (35)	30 (46.9)	0.18
Anti-platelet use (%)	30 (50)	29 (45.3)	0.73
Lipid-lowering therapy use (%)	50 (83.3)	47 (73.4)	0.18
Anti-hypertensive use (%)	50 (83.3)	53 (82.8)	0.94
Severity score 1 (%)	8 (13.6)	3 (4.8)	0.20
Severity score 2 (%)	21 (35.6)	19 (30.6)	
Severity score 3 (%)	9 (15.3)	8 (12.9)	
Severity score 4 (%)	21 (35.6)	32 (51.6)	
Using insulin therapy (%)	48 (80)	53 (82.8)	0.97

### Baseline factors affecting mortality

In order to ascertain which baseline characteristics were impactful on mortality, variables were added individually to a Cox-regression model with HR, 95% CI and *p*-values calculated. Diabetes Severity Score, insulin use and age were found to significantly impact mortality, whereas established cardiovascular disease at baseline; duration of diabetes; baseline HbA1c; presenting capillary blood glucose; and anti-platelet, anti-hypertensive and statin therapies failed to show an effect. Data are displayed in Table 2.

**Table 2.** The impact of baseline characteristics on mortality following severe community hypoglycaemia. Data in bold represent significant differences.

Variable	Crude hazard ratio (HR) and 95% confidence interval (CI)	*p*-value	Adjusted hazard ratio (HR) and 95% confidence interval (CI)	*p*-value
Gender (female)	1.11 (0.64–1.92)	0.71	—	—
Age (years)	**1.04 (1.011 0.08)**	**< 0.01**	1.03 (0.99–1.07)	0.13
HbA1c (mmol/mol)	1.01 (0.99–1.03)	0.59	—	—
Group (intensive vs standard)	0.60 (0.34–1.06)	0.08	0.68 (0.38–1.19)	0.18
Duration of diabetes (years)	1.01 (0.99–1.03)	0.45	—	—
Presence of established cardiovascular disease (yes/no)	1.32 (0.77–2.28)	0.31	—	—
Anti-platelet use (yes/no)	1.25 (0.61–2.58)	0.54	—	—
Lipid-lowering therapy use (yes/no)	1.30 (0.65–2.58)	0.46	—	—
Anti-hypertensive use (yes/no)	1.34 (0.61–2.98)	0.47	—	—
Diabetes Severity Score (groups 3+4 vs 1+2)	**4.23 (2.15–8.60)**	**< 0.001**	**3.63 (1.78–7.30)**	**< 0.001**
Insulin use (yes/no)	**2.43 (1.04–5.71)**	**0.04**	1.91 (0.78 – 4.71)	0.16

### Comparison between study groups

Overall, the mortality rate was 1.5-fold greater in the standard care arm as compared to the intervention arm with 32 (50%) and 20 (33%) deaths occurring, respectively. Using a Cox-regression model, a comparison between groups was made after adjustment for factors shown to correlate with mortality (age, diabetes severity score and insulin use), with HR = 0.68 (95% CI: 0.38–1.19, *p* = 0.18) in favour of the intervention (Table 2). After adjustment for additional covariates, HR was largely unchanged at 0.71 (95% CI: 0.35–1.43).

**Figure 1.**
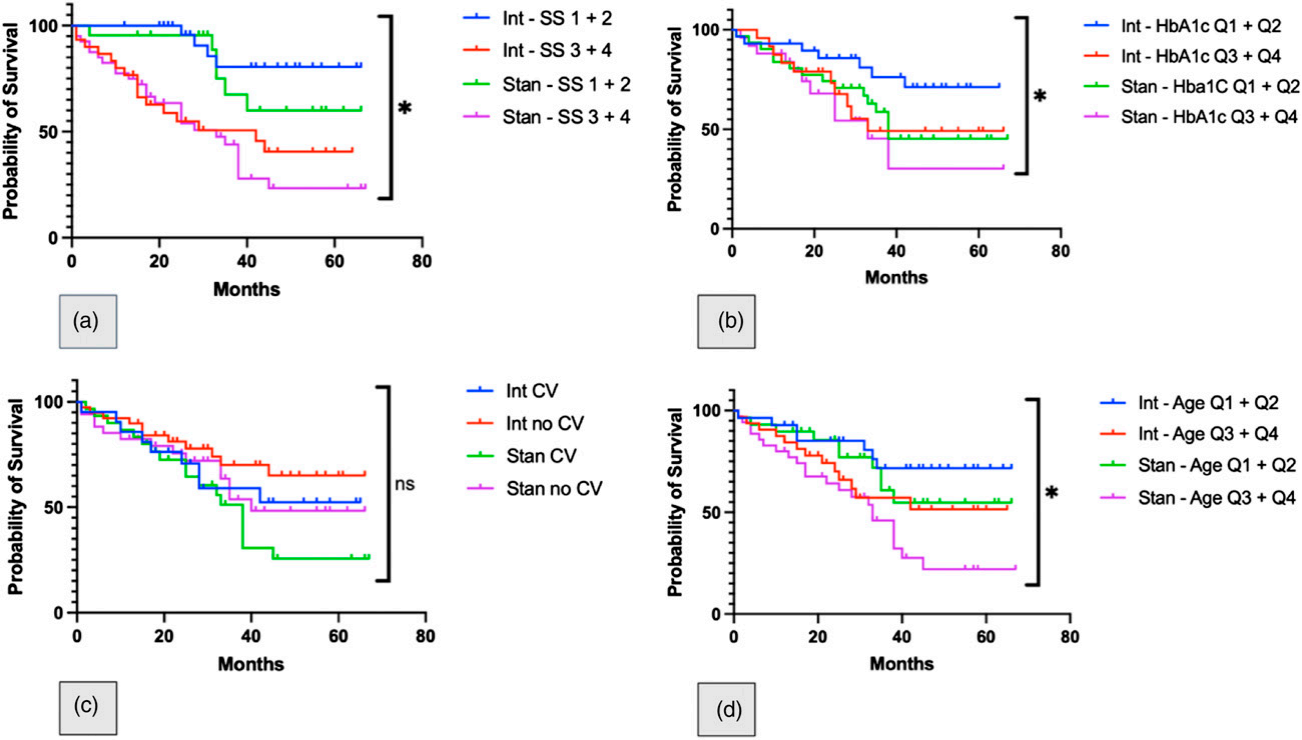
Survival curves comparing different groups. (a) Spilt by Diabetes Severity Score (DSS), (b) split by baseline HbA1c quartiles, (c) split by presence of established cardiovascular disease (CV) at baseline and (d) split by age quartile. Int = intervention; Stan = standard. * denotes *p* = <0.05 for the logrank test for trend.

We also attempted to demonstrate the benefit of the intervention in subgroups of participants. Four variables were selected (baseline HbA1c, baseline Diabetes Severity Score, presence of established cardiovascular disease at baseline and age at baseline) and Kaplan-Meier curves were constructed to depict differences in mortality between study groups. In order to depict differences, severity scores 1 + 2 and 3 + 4 were grouped together as were quartiles of age and baseline HbA1c. A logrank test was used to compare trends between curves, with significance shown for severity score, HbA1c quartile and age quartile. This data is displayed in Figure 1.

### Cause of death

We studied cause of death across the two groups using death certificates, and full details can be found elsewhere.^9^ The main cause of death in the whole group was infections, followed by cardiovascular disease. There was a clear difference in cardiovascular death, comparing the two study arms with a single cardiovascular death occurring in the intervention arm (1.7%) vs 12 (18.8%) in the standard arm (*p* = 0.002). The intervention had no effect on other causes of death including infection, renal disease or dementia.

## Discussion

Severe hypoglycaemia in those with type 2 diabetes necessitating the assistance of emergency services results in high mortality, which can be modulated following a simple and pragmatic intervention focussed on patient education, glucose monitoring and medication review. From this exploratory analysis, we show that some pre-treatment characteristics influence mortality following severe hypoglycaemia, yet the studied intervention was beneficial even following adjustment for such variables. Overall, single variables do not adequately predict which patients may benefit from intervention with classical markers of risk, such as HbA1c and the presence of established cardiovascular disease, failing to provide significant impact on mortality, albeit in a relatively small sample size. The strongest predictor of mortality was a composite Diabetes Severity Score which incorporates hard clinical endpoints and biochemical data from individuals.

These results highlight the significant risk to those with type 2 diabetes following severe hypoglycaemia; the benefit of a relatively straightforward and non-invasive intervention; and raise the question as to whether diabetes severity score, calculated through a validated scoring system, should be incorporated into the management of such patients, prioritising higher risk individuals. Our findings extend recent work investigating the impact of multimorbidity on hypoglycaemia risk^11^ and suggest the potential utility of severe hypoglycaemia risk stratification for assessing treatment response to subsequent intervention.

Importantly, this exploratory analysis suggests that the intervention is beneficial in all study participants, regardless of baseline characteristics, although statistical significance was not always reached due to small sample size. Given the small sample size and the fact that this was a single centre study, larger multicentre studies are urgently needed to provide more robust data that can be incorporated into national guidelines in order to improve outcome in this high-risk group of patients with severe hypoglycaemia.

These data add to the existing literature in the field and also provide reassurance that the intervention is beneficial across a wide range of patients and is not limited to certain subgroups. In particular, the reduction in cardiovascular mortality in the intervention arm of the study carries an important clinical message and emphasises the need for adequate glycaemic support in the older population with diabetes. However, it should be acknowledged that this work was not a definitive outcome trial but supports the need for further research in this field through large multicentre studies with the ultimate aim of optimising care in this vulnerable group of individuals with diabetes.

A strength of this study is the uniformity of patient care, the completeness of data regarding baseline characteristics and the simplicity of study intervention. There are a number of drawbacks that should be acknowledged, including small sample size and single geographical location, preventing generalisation to different healthcare systems and different ethnic groups, particularly as > 80% of participants were of Caucasian ethnicity. There are also numerical differences in study groups despite randomisation, namely, a higher percentage of participants in the standard care group who had established cardiovascular disease and who had the highest diabetes severity score of 4, although higher BMI and longer diabetes duration in the intervention group may have balanced out the risks.

## Conclusions

For patients with T2D sustaining an episode of severe hypoglycaemia in the community, a structured intervention appears to be helpful in all patients regardless of their baseline risk factors and Diabetes Severity Score. However, those with higher diabetes severity score appear to be at particular risk and may require prioritisation of the intervention. A large multicentre randomised controlled trial is urgently needed to provide conclusive data that a similar intervention should be part of routine clinical practice.

## Supplemental Material

sj-pdf-1-dvr-10.1177_14791641211067415 – Supplemental Material for Risk factors associated with mortality in individuals with type 2 diabetes following an episode of severe hypoglycaemia. Results from a randomised controlled trial Click here for additional data file. Supplemental Material, sj-pdf-1-dvr-10.1177_14791641211067415 for Risk factors associated with mortality in individuals with type 2 diabetes following an episode of severe hypoglycaemia. Results from a randomised controlled trial by Sam M Pearson, Noppadol Kietsiriroje, Beverley Whittam, Rebecca J. Birch, Matthew D Campbell and Ramzi A Ajjan in Diabetes and Vascular Disease Research
