# Supplementary material for: Risk factors associated with mortality in individuals with type 2 diabetes following an episode of severe hypoglycaemia. Results from a randomised controlled trial
Source: Diab Vasc Dis Res. 2022 Jan 28;19(1):14791641211067415. doi: 10.1177/14791641211067415 (PMC8801660; doi:10.1177/14791641211067415)
Supplement: sj-pdf-1-dvr-10.1177_14791641211067415 – Supplemental Material for Risk factors associated with mortality in individuals with type 2 diabetes following an episode of severe hypoglycaemia. Results from a randomised controlled trial [file sj-pdf-1-dvr-10.1177_14791641211067415.pdf]

Supplementary 1. Revised type 2 diabetes vascular staging instrument with detailed classification items and their measures

Vascular stage & broad classification of stages

Detailed classification item	Measures (Australian Refined Diagnosis Related Group code version 5.0[*] or clinical reference range)
1 Diagnosis of type 2 diabetes	Doctor diagnosis confirmed by a medical chart audit or a fasting blood glucose level ≥ 7 mmol/L[†] or 2-hour glucose tolerance test result of blood glucose level ≥ 11.1 mmol/L[†][10] or a hospital admission for diabetes
With no evidence of microvascular or macrovascular risk factors (but may be obese or report current tobacco smoking)	
Albumin creatinine ratio (ACR) within normal range	ACR of 0–2.5 mg/mmol for men and 0–3.5 mg/mmol for women[(]
Normal high density lipoprotein cholesterol (HDLC)	> 1.0 mmol/L[)]
Normal triglycerides	< 1.5 mmol/L[)]
Not hypertensive	Not hospitalised for hypertension (DRG code F67A or F67B), or blood pressure < 140/90 mmHg[‖]
No evidence of retinopathy	Not hospitalised for retinal procedures and vascular disorders of the eye (DRG code C03Z or C16Z)
Peripheral sensation normal and no evidence of claudication	Not hospitalised for DRG code F65A or F65B
No cardiac disease	Not hospitalised for DRG code A05Z, F12Z, F14A, F14B, F62Z, F62B, F70A, F70B, F62A, F14C, F15Z, F16Z, F17Z, F18Z, F19Z, F10Z, F41A, F41B, F60A, F60B or F60C
2 Screen-detected microvascular comorbidities and/or risk factors for macrovascular disease	
Microalbuminuria	ACR 2.6–25 mg/mmol for men and 3.6-35 mg/mmol for women[(]
Reduced HDLC	≤ 1.0mmol/L[)]
Elevated triglycerides	≥ 1.5mmol/L[)]
Hypertension	Hospitalised for DRG code F67A or F67B, or systolic blood pressure ≥140 mm Hg and/or a diastolic blood pressure ≥ 90 mm Hg[‖]
3 Moderate microvascular and/or macrovascular comorbidities	
Macroalbuminuria	ACR > 25 mmol/L for men and > 35 mmol/L for women[(]
History of coronary artery bypass graft or angioplasty	F05A, F05B, F06A, F06B
History of carotid artery disease	F66A, F66B
History of or hospitalised for angina	
History of claudication or peripheral vascular disease	
4 Late-stage diabetes macrovascular and/or microvascular comorbidities	
Amputation of lower limb	F11A, F11B, F13Z, I07Z, I14Z
Present or history of gangrene of the lower limb	J13A, J13B, J64A, J64B
History of stroke with residual deficit	B69A, B69B, B70A, B70B, B70C, B70D
History of or hospitalised for cardiac failure	A05Z, F12Z, F14A, F14B, F62A, F62B, F70A, F70B
History of myocardial infarction	F14C, F15Z, F16Z, F17Z, F18Z, F19Z, F10Z, F41A, F41B, F60A, F60B, F60C
Proliferative retinopathy and both eyes: only able to perceive light or hand movement or count fingers, or unable to perceive light	C03Z, C16Z
Present or history of osteomyelitis or ulcer of the foot or charcot joint	I64A, I64B
Bacterial soft tissue infection of the foot	J12A, J12B, J12C,
History of painful neuropathy or autonomic neuropathy other than erectile impotence	K01Z
History of end-stage renal failure	L60A, L60B, L60C
History of dialysis	L61Z
History of renal transplant	A09A, A09B

Sources:

[*] Department of Health and Aging: Public Sector - Estimated Round 9 (2004–05) AR_DRG 5.0 Cost Report. [http://www.health.gov.au/internet/main/publishing.nsf/Content/88F4E78E15620A80CA2571CB0004DDAA/$File/_R9CWQLDEst.pdf]

[(] Diabetes Australia's Health Care and Education Committee and RACGP's National Standing Committee for Quality Care in 2010: Diabetes management in general practice. [http://www.racgp.org.au/Content/NavigationMenu/ClinicalResources/RACGPGuidelines/Diabetesmanagement/200910diabetesmanagementingeneralpractice.pdf]

[‖] National Blood Pressure Advisory Committee: Hypertension management guide for doctors 2004. [http://www.sld.cu/galerias/pdf/servicios/hta/hypertension_management_guide_australia_2004.pdf]

[)] National Heart Foundation of Australia and Cardiac Society of Australia and New Zealand: Lipid management guidelines-summary paper. *Medical J Aust* 2001, 175: S57-S88.

[†] World Health Organization: Part 1: diagnosis and classification of diabetes mellitus, in: definition, diagnosis and classification of diabetes mellitus and its complications. [http://www.staff.ncl.ac.uk/philip.home/who_dmg.pdf]

Supplementary 1: The diabetes severity scoring system used in this work is taken from Gibson et al. BMC Health Services Research 2012, 12:185.
